# Association between gout and the development of Parkinson’s disease: a systematic review and meta-analysis

**DOI:** 10.1186/s12883-022-02874-0

**Published:** 2022-10-11

**Authors:** Asra Fazlollahi, Mahdi Zahmatyar, Hossein Alizadeh, Maryam Noori, Nasrin Jafari, Seyed Aria Nejadghaderi, Mark J. M. Sullman, Koroush Gharagozli, Ali-Asghar Kolahi, Saeid Safiri

**Affiliations:** 1 Student Research Committee, Tabriz University of Medical Sciences, Tabriz, Iran; 2 School of Medicine, Shahid Beheshti University of Medical Sciences, Tehran, Iran; 3 Student Research Committee, School of Medicine, Iran University of Medical Sciences, Tehran, Iran; 4 Urology Research Center, Tehran University of Medical Sciences, Tehran, Iran; 5 Department of Epidemiology and Biostatistics, Faculty of Health, Tabriz University of Medical Sciences, Tabriz, Iran; 6 Research Center for Integrative Medicine in Aging, Aging Research Institute, Tabriz University of Medical Sciences, Tabriz, Iran; 7 Systematic Review and Meta-Analysis Expert Group (SRMEG), Universal Scientific Education and Research Network (USERN), Tehran, Iran; 8 Department of Life and Health Sciences, University of Nicosia, Nicosia, Cyprus; 9 Department of Social Sciences, University of Nicosia, Nicosia, Cyprus; 10 Brain Mapping Research Center, Shahid Beheshti University of Medical Sciences, Tehran, Iran; 11 Social Determinants of Health Research Center, Shahid Beheshti University of Medical Sciences, Tehran, Iran; 12 Neurosciences Research Center, Aging Research Institute, Tabriz University of Medical Sciences, Tabriz, Iran; 13 Department of Community Medicine, Faculty of Medicine, Tabriz University of Medical Sciences, Tabriz, Iran

**Keywords:** Gout, Uric acid, Parkinson's disease, Hyperuricemia, Systematic review, Meta-analysis

## Abstract

**Background:**

As a natural antioxidant, uric acid plays a protective role against neurodegenerative disorders, including Parkinson’s disease (PD). Therefore, the risk of PD has been found to be lower in people with hyperuricemia. In this article, we conducted a systematic review and meta-analysis to investigate whether gout affects the future risk of developing PD.

**Methods:**

We searched PubMed, Scopus, the Web of Science, and Google Scholar to find relevant studies, up to March 16, 2022. Studies investigating the risk of PD, following a gout diagnosis, were included if they were cross-sectional, case–control or cohort studies. The Newcastle Ottawa Scale (NOS) checklist was used to assess the quality of all included studies. The meta-analysis was performed using STATA 17.0.

**Results:**

Ten studies were included, which were comprised of three case-controls, six cohort studies and one nested case–control study. We found no significant association between gout and the risk of PD among both sexes (RR = 0.94, 95% CI: 0.86–1.04), although the association was significant for females (RR = 1.09; 95% CI: 1.02–1.17). Subgroup analysis also showed no significant findings by age group, whether they were receiving treatment for gout, study design, quality assessment score, and method of gout ascertainment. In contrast, the studies that defined PD according to the use of drugs showed significant results (RR = 0.82; 95% CI: 0.76–0.89). There was a significant publication bias on the association between gout and PD.

**Conclusions:**

The presence of gout had no significant effect on the risk of subsequently developing PD. Further analyses are recommended to investigate the effects of demographic and behavioral risk factors.

**Supplementary Information:**

The online version contains supplementary material available at 10.1186/s12883-022-02874-0.

## Introduction

Parkinson’s disease (PD) is a chronic, progressive disease primarily characterized by the degeneration of dopaminergic nigrostriatal neurons [1–3]. PD is the second most common neurodegenerative disease, after Alzheimer’s disease [4–8]. The prevalence of PD is 0.3% among the general populations of industrialized countries, 1% in the population over 60 years old and 4% in the population over 80 years old [4, 8]. Several factors are likely implied in the pathophysiology of PD and ultimately in dopaminergic neuron loss and alpha-synuclein pathology [6, 7]. Evidence suggests that oxidative stress and free radical production play a critical role in PD pathogenesis [1, 9–13]. Additional factors, such as mitochondrial dysfunction, neuro-inflammation, and excitatory toxicity, may also contribute to neuronal cell damage in PD [14–16]. Perturbation of mitochondria homeostasis and production of reactive oxygen species may result from environmental factors or mutations in specific genes, such as the Leucine-Rich Repeat Kinase 2 (LRRK2) gene, or a combination of the two [14, 17]. Dopamine plays a fundamental role in the modulation of motor control and output of smooth and balanced movements [18]. Reduction in dopamine levels in PD results in symptoms such as bradykinesia, rigidity, resting tremors, and postural imbalance, which become manifest when 70–80% of dopamine-producing neurons are lost [1, 19, 20].

The intracellular metabolism of dopamine is prone to the production of free radicals, therefore exposing dopaminergic cells to a greater risk of oxidative stress toxicity [21]. Urate or uric acid, as a metabolite of purine, is a powerful natural antioxidant that has an important role in eliminating free radicals [7, 14, 19, 22, 23]. With this in mind, it has long been debated whether people who have higher levels of urate are at a lower risk of developing PD [7, 14, 22–25]. It should be noted that, apart from a lower risk of PD, high levels of urate in serum and cerebrospinal fluid have been associated with a slower rate of clinical progression [7, 14, 24, 25]. However, definitive mechanisms of action for urate as a neuro-protection substance have yet to be determined [26].

Since gout is a chronic form of hyperuricemia, there is a possibility that gout may have a protective effect on the development of PD. Therefore, the present systematic review and meta-analysis aimed to evaluate whether an association exists between gout and the subsequent onset of PD.

## Methods

We performed this systematic review and meta-analysis in accordance with the Preferred Reporting Items for Systematic Reviews and Meta-Analyses (PRISMA) guidelines [27].

### Search strategy

In the present study, two authors independently searched the electronic databases, including PubMed, Scopus and the Web of Science to find relevant studies from the inception of the databases up to March 16, 2022. Furthermore, we manually searched the first 100 pages of the Google Scholar search engine to identify any eligible studies. There were no limitations or restrictions in the search fields, such as study type, date, or language. We also used backward and forward citation searches of the included studies, in order to find as many relevant articles as possible. Our search strategy included all terms related to Gout and PD, which is described in detail in Supplementary Table 1.

### Study selection

Two researchers independently screened the title and abstract of all identified articles, based on the eligibility criteria. Following this, the same researchers reviewed the full texts of all selected articles. The inclusion criteria were as follows: 1) Cross-sectional studies investigating the development of PD in subjects with gout; 2) Case–control or cohort studies comparing the risk of PD in participants with and without gout; 3) odd ratio (OR), hazard ratio (HR), or relative risk (RR) with 95% confidence interval (CI) were reported or could be calculated. We excluded those studies which included patients diagnosed with PD before being diagnosed with gout. In addition, case reports, case series, editorials, commentaries, letters, review articles, notes, news, book chapters, meta-analyses, and the re-analysis of previously published articles were excluded. Any disagreements between the two researchers were resolved by discussion or consultation with a third reviewer.

### Data extraction

We conducted the data extraction process using predefined Microsoft Office Excel forms. Two authors independently collected the following information from each eligible study: first author, study title, year of publication, country of study, sample size, study population, age range of participants, follow-up duration, smoking status, status of anti-gout therapy, using caffeine or diuretic agents or non-steroidal anti-inflammatory drugs (NSAIDs) or other drugs that can play a role in the treatment or augment the risk of gout disease (e.g., Allopurinol, Colchicine, Tacrolimus, Cyclosporine, Probenecid, beta blockers, angiotensin converting enzyme inhibitors), comorbidities, diet status, Charlson-Romano comorbidity score, gout diagnosis method, PD ascertainment, and the reported effect sizes (OR, HR, and RR), along with their 95% CIs that controlled for any potential confounders. Any discrepancies between the two researchers were resolved by discussion or consulting another reviewer.

### Quality assessment

To assess the risk of bias and study eligibility of the selected articles, two authors independently used the Newcastle Ottawa Scale (NOS) to evaluate and score each included study according to the different parameters [28]. In brief, this scale appraised the quality assessment of each study across three domains: selection of the participants for each group; the comparability between the study groups; and the ascertainment of exposure in the case-control study or the outcome of interest in a cohort study. A study can be awarded a maximum of one star for each numbered item within the selection, and exposure and outcome categories, while a maximum of two stars can be given for comparability. A third investigator resolved any disagreements between the two authors.

### Statistical analysis

The meta-analysis was performed using Stata 17.0 (Stata Corp, LLC, TX). We assessed statistical heterogeneity using the Q test and *I*^2^ statistics. I-square values above 50% represent significant heterogeneity. In the case of significant heterogeneity, we performed a random effect analysis, otherwise a fixed effect model was used [29, 30]. The reported HRs were considered to be equal to RRs [31]. ORs were considered to be equal to RRs, if the incidence rate in the included studies was low (< 10%) or the ORs were between 0.5 and 2.5. Otherwise we converted the ORs to RRs, based on the method proposed by Zhang et al. [32]. We aimed to use funnel plots for evaluating publication bias, if at least ten studies were included [33]. Subgroup analysis was performed according to age, gout treatment status, study design, methodological quality scoring, and disease definition. Moreover, the Egger’s test was used to evaluate publication bias [34]. A *p*-value of less than 0.5 was treated as statistically significant.

## Results

**Fig. 1:**
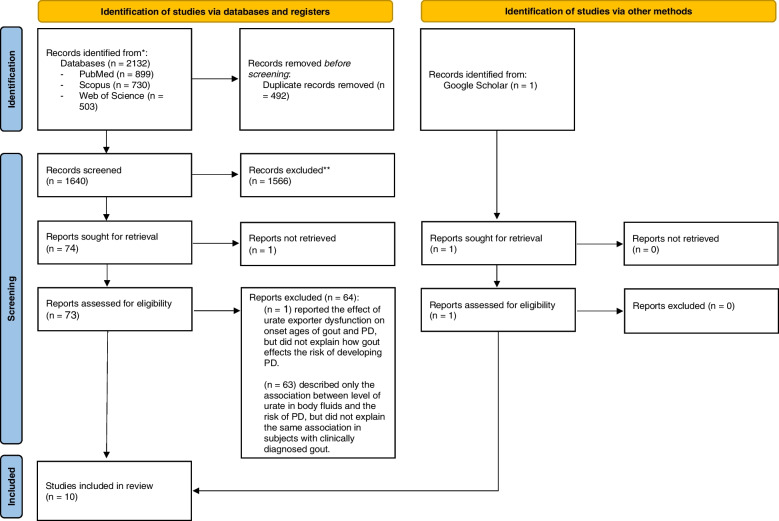
Study selection process

A total of 2,133 articles were identified from PubMed (*n* = 899), Scopus (*n* = 730), the Web of Science (*n* = 503), and Google Scholar (*n* = 1). Following the removal of 492 duplicate articles, the remaining 1,641 studies were screened and 75 records were selected for full text review. Sixty four publications were excluded because they did not report the outcome of interest. We could not access the full text of one article and thus it was also excluded [35]. The remaining ten records were used for qualitative and quantitative synthesis [36–45] (Fig. 1).

### Study characteristics

Three studies were case-controls [38, 39, 45], one was a nested case–control [36], and six were retrospective cohort studies [37, 40–44]. The included articles were published between 2007 and 2022 and all were written in English. A total of 15.3 million participants were included in the meta-analysis, with 790,643 cases and about 14.5 million in the control group. Two studies reported data from the United Kingdom [36, 40], two from Taiwan [39, 43], and there was one study from each of the following countries: Canada [37], Denmark [38], Korea [44], Norway [41], the United States [42], and Spain [45]. Five studies reported that the cases used anti-gout therapies, like allopurinol, probenecid, and colchicine [36–38, 41, 44], but the level of urate and dietary patterns were not reported by any of the included articles. The comorbid conditions of participants are summarized in Supplementary Table 2. Gout diagnosis was based on clinical criteria in eight studies [36, 37, 39, 40, 42–45], while two studies based the diagnosis of gout on the concomitant use of anti-gout medications [38, 41]. Concerning diagnosis of PD, two studies diagnosed PD based on the use of therapeutic drugs (i.e. levodopa) [41, 45], while the rest used clinical criteria [36–40, 42–44]. Table 1 shows the characteristics of the included studies.

**Table 1:** Baseline characteristics of studies included in the meta-analysis

Study ID	Country	Study Design	Follow-up period	Study Population	Sample Size	Case, n	Control, n	Mean age (SD)	Male (%)	Gout Ascertainment	PD Ascertainment	Receiving Anti-Gout Therapy
Alonso et al. 2007 [36]	UK	Nested case–control	Start: January 1, 1995, or after 3 years of continuous recorded medical history, whichever came later end: first computerized PD symptom (tremor, rigidity, bradykinesia, abnormal gait), PD diagnosis, use of drugs that may cause parkinsonism, last data collection, death, or December 31, 2001, whichever came first	Britons whose information is recorded in the General Practice Research Database (GPRD)	7,686	1,052	6,634	Case: 70.0 (9.4) Control: 68.7 (9.1)	Case: 632 (60.1) Control: 3,921 (59.1)	Computerized gout diagnoses recorded in the GPRD -treated gout: the presence of a gout diagnosis plus at least one anti-gout prescription before the index date -untreated gout: no prescriptions associated with the gout diagnosis	Computer-recorded PD diagnosis and at least two prescriptions to treat parkinsonian signs	Yes (the treated group)
Cortese et al. 2018 [41]	Norway	Retrospective cohort	From 01/01/2005 to PD onset, emigration, death, or end of follow-up on 31/12/2013	The whole Norwegian population alive and at least 18 years old on 1st January 2004	3,572,437	108,520	3,463,917	Case: 62.1 Control: 46.3	Case: 71,885 (66.2) Control: 1,690,259 (48.8)	Use of urate-lowering drugs, including allopurinol, probenecid, and colchicine; from the Norwegian Prescription Database	Receiving at least 365 DDDs of levodopa (ATC: N04BA) according to NorPD along with a diagnostic code for PD	Yes (the only exposure)
De Vera et al. 2018 [37]	Canada	Retrospective cohort	An 8-year median follow-up period	The entire population of the province of BC whose information is recorded in the BC Linked Health Database (BCLHD)	67,457	11,258	56,199	Case: 74.1 (6.5) Control: 74.1 (6.5)	Case: 7,482 (66.5) Control: 37,330 (66.4)	2 visits at least 1 day apart with the International Classification of Diseases, Ninth Revision (ICD-9) code of 274	The first recorded event of diagnosis for PD using the ICD-9 code for PD, 332 or At least 2 prescriptions of anti-Parkinsonian medications	Yes (72% of cases)
Hu et al. 2020 [43]	Taiwan	Retrospective cohort	Since the enrollment date until (1) PD diagnosis by a neurologist, (2) death, or (3) the end date, December 31, 2013	Taiwan residents whose information is presented in Longitudinal Health Insurance Database 2000 (LHID2000)	15,800	7,900	7,900	Case: 50 Control: 50	Case: 5,409 (83.9) Control: 5,409 (83.9)	Diagnosing according to ICD-9-CM code 274 plus having at least two consensus diagnoses of gout during the observational period	Neurologist-diagnosed PD	N/A
Kim et al. 2021 [44]	Korea	Retrospective cohort	N/A	Almost all Korean individuals enrolled in the NHIS database	654,320	327,160	327,160	N/A	Case: 304,162 (93) Control: 304,162 (93)	Diagnosis of gout (ICD-10, M10) who were prescribed medications for gout, such as colchicine, allopurinol, febuxostat, and benzbromarone for at least 90 days	Assigning a diagnosis code (ICD G20) and registering in the rare incurable diseases (RID) system	Yes (the only exposure)
Lai et al. 2014 [39]	Taiwan	case–control	N/A	All Taiwanese insured residents whose data is presented in the National Health Research Institute in Taiwan (NHRI)	19,270	3,854	15,416	Case: 75.0 (5.0) Control: 74.0 (5.3)	Case: 1,994 (51.7) Control: 7,976 (51.7)	Diagnostic code of the Taiwan National Health Insurance Database	Diagnostic codes of the Taiwan National Health Insurance Database	N/A
Pakpoor et al. 2015 [40]	UK	Retrospective cohort	N/A	People admitted to hospital day case care or inpatient care in all English National Health Service (NHS) hospitals	> 9 million	214,653	∼ 9 million	N/A	Case: 158,200 (73.7) Control: N/A	Diagnosis of gout in an episode of hospital care by identifying the first episode of day case care, or admission for gout	Diagnostic code of the database plus the English National Death Registration	N/A
Schernhammer et al. 2013 [38]	Denmark	Case–control	N/A	Patients registered in nationwide Danish in- and outpatient Hospital Register records	26,900	4,484	22,416	N/A	Case: 2,676 (59.7) Control: 13,378 (59.7)	At least one prescription of anti-gout drugs	First time diagnosis of PD between 2001–2008 and a diagnosis confirming PD medication history according to the Danish National Prescription Registry (DNPR)	Yes (the only exposure)
Singh et al. 2019 [42]	USA	Retrospective cohort	N/A	Individuals registered in Centers for Medicaid and Medicare (CMS) Chronic Condition Data Warehouse	1,725,833	94,133	1,631,700	N/A	N/A	Diagnosis of gout (International Classification of Diseases-10 (ICD 10), M10) and prescribing medications for gout, including colchicine, allopurinol, febuxostat, and benzbromarone for at least 90 days	The new occurrence of at least two claims for PD at least 4 weeks apart, identified by the presence of an International Classification of Diseases, ninth revision, common modification (ICD-9-CM) diagnostic code, 332.xx, with no previous diagnostic code for PD in the baseline 365-day period	Yes (the treated group)
Pou et al. 2022 [45]	Spain	Case–control	A 20-year median follow-up period	Patients whose information is presented in the public primary care health system of the city of Barcelona	88,145	17,629	70,516	Case: 75.76 (11.07) Control: 75.13 (11.1)	Case: 7,815 (44.3) Control: 31,260 (44.3)	Retrospective clinical record	A new diagnosis register of PD, or a new prescription of dopaminergic drugs (levodopa, dopaminergic agonists, amantadine, selegiline or rasagiline), between 2010 and 2019	N/A

### Overall analysis of the association between gout and PD

**Fig. 2:**
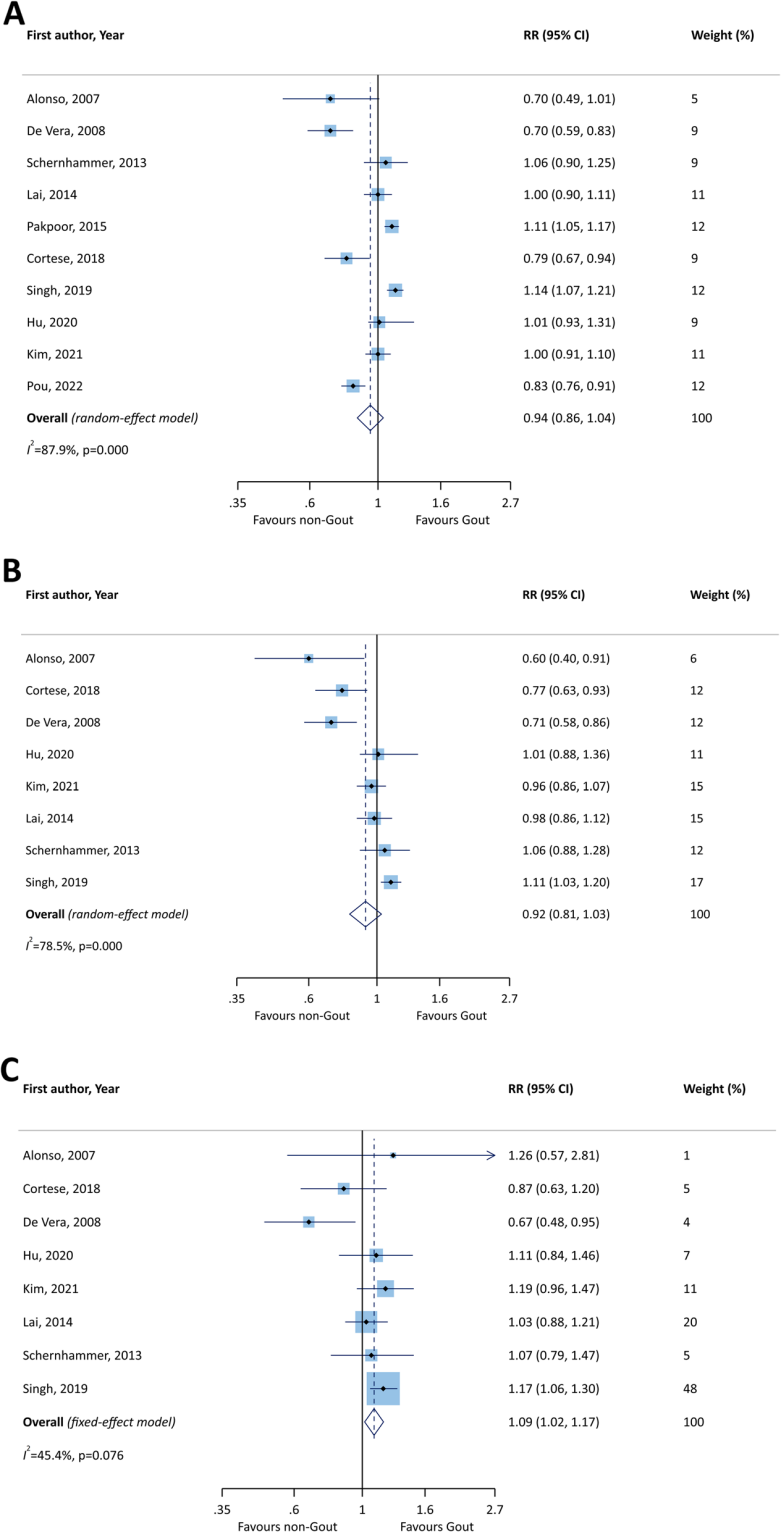
Forest plots of the association between gout and Parkinson’s disease in both sexes (A), males (B), and females (C). RR: relative risk; CI: confidence interval

There was no significant association between gout and the subsequent development of PD (RR = 0.94, 95% CI: 0.86 to 1.04) (Fig. 2A). Moreover, the RRs of PD development in patients with gout were 0.92 (95% CI: 0.81 to 1.03) and 1.09 (95% CI: 1.02 to 1.17), among males and females respectively (Figs. 2B and Fig. 2C).

### Subgroup analysis

**Fig. 3:**
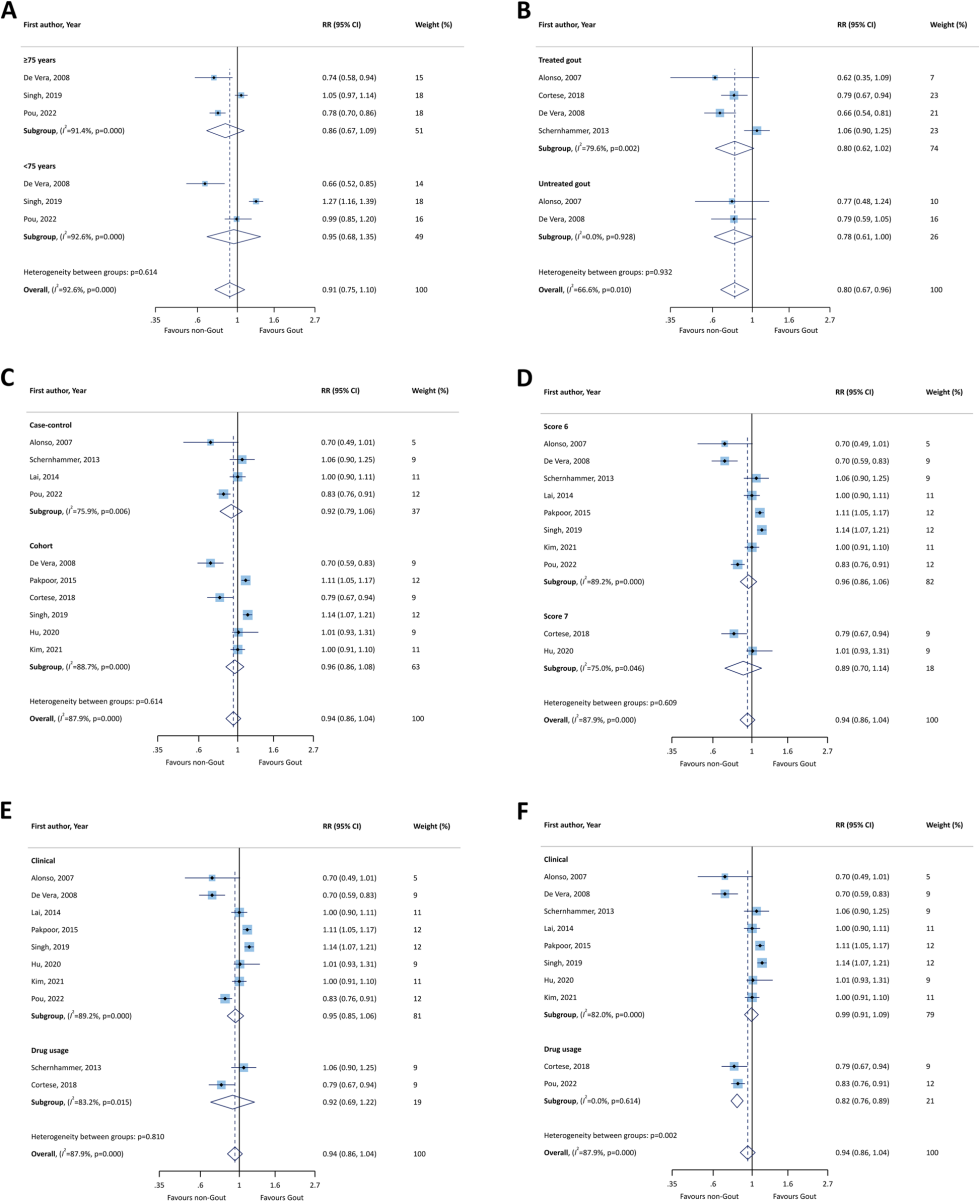
Forest plots of the association between gout and Parkinson’s disease by age (A), treatment status for gout (B), study design (C), quality assessment scores (D), gout ascertainment (E), and Parkinson’s disease ascertainment (F). RR: relative risk; CI: confidence interval

Subgroup analysis was undertaken comparing age group, whether patients were receiving treatment for gout, study design, quality assessment scores, and type of gout and PD diagnoses. The pooled RRs for the age groups < 75 years old and ≥ 75 years of age were 0.95 (95% CI: 0.68 to 1.35) and 0.86 (95% CI: 0.67 to 1.09), respectively (Fig. 3A), and therefore not statistically significant. In terms of whether or not they were receiving treatment for gout, the treated patients had a higher RR than those who received no treatment (RR = 0.80; 95% CI: 0.62 to 1.02 vs. RR = 0.78; 95% CI: 0.61 to 1.00) (Fig. 3B), although this did not reach statistical significance. The subgroup analysis by study design showed no significant difference between the cohorts and case–control studies in the association between gout and PD (RR = 0.96; 95% CI: 0.86 to 1.08 for cohorts and RR = 0.92; 95% CI: 0.79 to 1.06 for case-controls) (Fig. 3C). However, studies with a quality assessment score of six (RR = 0.96; 95% CI: 0.86 to 1.06) had higher effect sizes than those with a score of seven (RR = 0.89; 95% CI: 0.70 to 1.14) (Fig. 3D), but this was not statistically significant. Studies which used clinical criteria for gout ascertainment had higher RRs than those which diagnosed gout based on the use of therapeutic drugs (RR = 0.95; 95% CI: 0.85 to 1.06 vs. RR = 0.92; 95% CI: 0.69 to 1.22, respectively), although both were non-significant (Fig. 3E). Finally, only two studies used drug administration to ascertain PD and these were statistically significant (RR = 0.82; 95% CI: 0.76 to 0.89) (Fig. 3F).

### Risk of bias assessment

The mean risk of bias score was 6.3, which ranged from 6 to 7. Out of six cohort studies, three had an overall score of seven [37, 41, 43] and the remaining three had scores of six [40, 42, 44]. The adequacy of the follow-up duration and the assessment of outcomes were the lowest scoring criteria (Supplementary Table 3). All case–control studies had a quality assessment score of six. Failing to report non-response rates and poor representativeness of the cases received the lowest scores (Supplementary Table 4).

### Publication bias

**Fig. 4:**
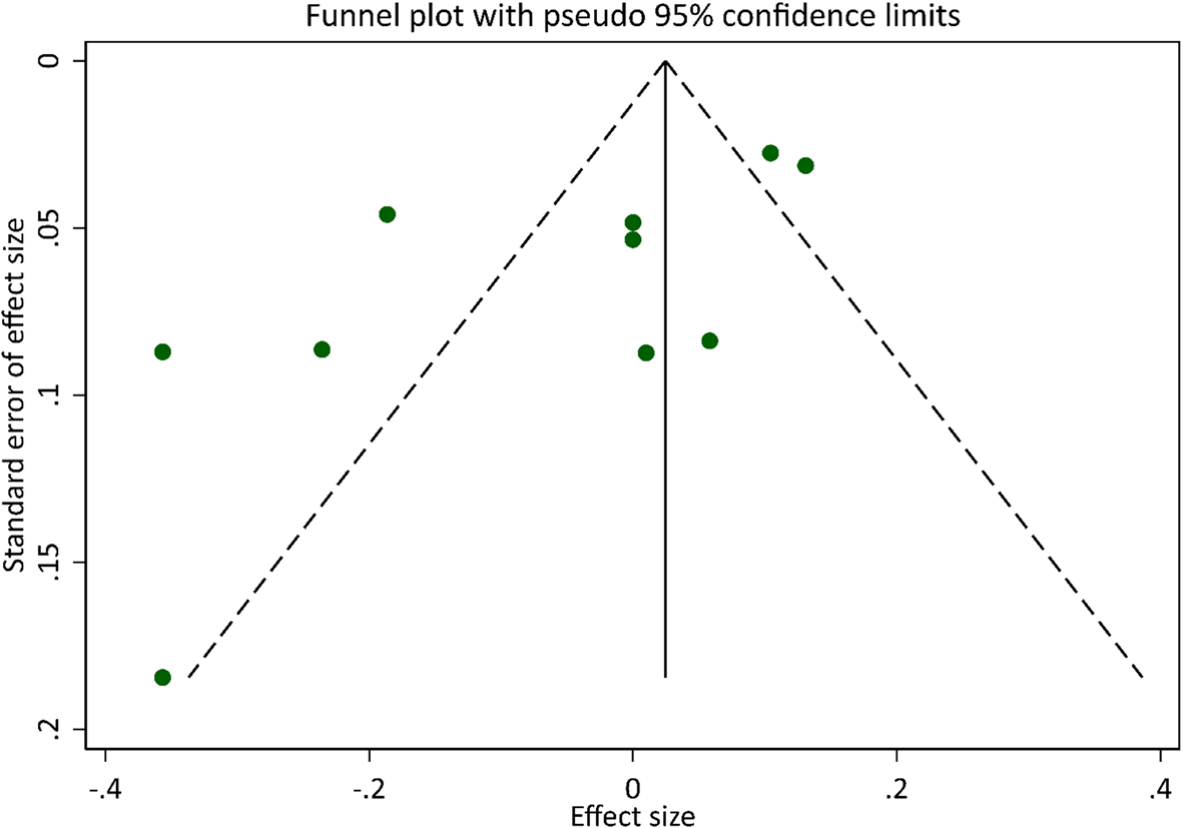
Funnel plot with 95% confidence limits for the association between gout and Parkinson’s disease

Egger’s test was statistically significant (p-value of 0.028), which indicates there was significant publication bias on the association between gout and PD. The funnel plot also shows an asymmetrical distribution of the included articles (Fig. 4).

## Discussion

The present systematic review and meta-analysis was conducted with the aim of examining whether an association exists between gout and the subsequent development of PD. The ten publications included approximately 800 thousand gout patients and nearly 14.5 million non-gout cases. The study did not find an inverse association between a history of gout and PD in either sex, which is in contrast with previous evidence showing a lower risk of developing PD in males with gout [36], or in both sexes [37]. Interestingly, and unexpectedly, the present study found a higher risk of developing PD in females with gout, therefore gout does affect the risk of PD, at least in this subgroup. Furthermore, none of the subgroup analyses by study design, methodological quality scoring, and the definition of gout were able to link the incidence of PD with a history of gout. The only subgroup that was significant was an inverse association found between gout and PD when PD ascertainment was made according to therapeutic drug use.

A previous meta-analysis, conducted in 2015, included three case–control and two cohort studies and aimed to investigate the possible association between gout and PD [46]. This review made similar findings to our own, in that the risk of PD did not change in accordance with a prior history of gout in the overall sample (RR = 0.93, 95% CI 0.79 to 1.09), as well as for male participants (RR 0.89, 95% CI 0.57 to 1.39). However, they also found no association between gout and PD among females (RR 0.95, 95% CI, 0.76 to 1.19), which was in contrast with our pooled estimates [46]. This difference might be a result of the model used for the data analysis, since the previous review used a random-effect model for all estimates, while we used a fixed-effect model for the female subgroup, since the estimated heterogeneity did not exceed the required threshold. Therefore, understanding sex differences in the association between gout and the later development of PD requires further large-scale cohort studies.

Another controversial issue is the predictive value of uric acid levels in the subsequent risk of PD development. A meta-analysis on nearly five thousand participants was performed with the objective of comparing the levels of serum uric acid in PD patients with those of a control group. The study came to the conclusion that serum uric acid levels were substantially lower in patients with PD, compared to the control group (standardized mean difference [SMD] = -0.49, 95% CI -0.67 to -0.30), for both male (SMD = -0.66, 95% CI -0.87 to -0.44) and female (SMD = -0.53, 95% CI -0.70 to -0.35) cases [47]. Furthermore, multiple prospective cohort studies have reported that uric acid may have a protective role in the pathogenesis of PD and may also reduce the rate of disease progression [48–50]. It has also been demonstrated that oxidative stress is a major contributor to the degeneration of dopaminergic neurons and the further development of PD [1, 9]. On the other hand, a natural antioxidative feature of uric acid was beneficial in the brain tissue through the uptake of urate by Glute-9 transporters in dopaminergic neurons [51, 52]. Thus, the neuroprotective effect of uric acid may be due to the scavenging of reactive oxygen and nitrogen species [53–55]. According to our findings, gout and hyperuricemia did not decrease the risk of PD onset, regardless of sex, but instead increased the risk among female patients. Therefore, while a consensus has been reached regarding the protective role of uric acid, the debate remains regarding the association between gout and PD.

The following points may explain this inconsistency. Firstly, it could be hypothesized that the protective role of uric acid on the development of PD follows a dose-gradient, in which gradually increasing the level of serum uric acid up to the upper limit of the normal range represents an inverse association with PD onset, while above that level the association disappears overall and a positive association appears among female participants. Secondly, given that the majority of patients with hyperuricemia never develop gout, there may be a genetic predisposing factor in which uric acid might lose the neuroprotective properties when a patient presents with symptoms of gout. Nevertheless, it could be argued that there may be more than one genetic factor, either leading to hyperuricemia (without gout) and lowering the risk of PD, or causing hyperuricemia with gout and not protecting from PD. Another possibility is that there is one genetic factor that reduces the risk of developing PD and is collaterally associated with hyperuricemia, but that hyperuricemia is an epiphenomenon that has no direct protective effect on dopaminergic neurons. Thirdly, the condition of pro-inflammation and oxidative stress, due to the deposit of monosodium urate in gout attacks [56], might offset the antioxidative benefit of uric acid. Fourthly, urate-lowering agents are more frequently administrated for symptomatic patients with gout, compared to hyperuricemic asymptomatic patients. Thus, these medications may interfere with the protective effects of uric acid. Fifthly, gout is a well-documented risk factor for the development of cardiovascular diseases, diabetes, and chronic kidney disease [57]. As a result, gout sufferers are at increased risk of premature death, which may decrease the number of individuals reaching the peak ages for the development of PD. On the other hand, cardiovascular risk factors are associated with vascular parkinsonism, an extrapyramidal motor syndrome that mimics PD and could therefore be misdiagnosed. This highlights the importance of using accurate diagnostic criteria to select subjects and minimize biases of the analyses. In this respect, the only two studies that found a statistically significant lower risk of developing PD in patients with gout are those that ascertained PD according to the use of dopaminergic therapy: a good response to levodopa is a reliable predictor of PD diagnosis accuracy, meaning that these studies likely selected a good case population.

Apart from acting as an antioxidant molecule, a paradoxical pro-oxidative role has also been linked to uric acid [58]. By increasing the production of free radicals, uric acid has been proposed to induce inflammatory reactions [59]. The free radicals mainly target lipids and membranes, instead of other cellular components. The presence of unsaturated fatty acids in the lipid structure of neuronal membranes renders neurons highly vulnerable to lipid peroxidation. Therefore, it might be that high levels of uric acid have a dual-effect, depending on the time of exposure. In this regard, uric acid may exert its protective effect during the initial stages of the elevated levels, primarily due to its antioxidative features, but following long-term exposure to these elevated levels, after the diagnosis of gout, it may induce an inflammatory reaction that damages the brain tissue [60].

Under normal conditions, the leakage of uric acid molecules through the blood brain barrier is low. However, it has been shown that the concentration of uric acid is higher in the cerebrospinal fluid of males, than among females [61]. In addition, a recent investigation showed that uric acid might increase oxidative stress by increasing the production of reactive oxygen species, without affecting the scavenging process, particularly among females [62]. Therefore, female gout patients are less likely to benefit from the neuro-protective advantages of uric acid in the brain tissue and more likely to be exposed to the harmful oxidative metabolites of uric acid, compared to males. This results in the higher susceptibility of female patients to PD onset, as suggested by our findings.

7The present meta-analysis has several advantages over the earlier attempt [46]. Firstly, this is the most up-to-date meta-analysis examining the association between gout and the risk of subsequent PD development. Secondly, five large scale studies were included in our meta-analysis, which makes the final conclusions more robust. Thirdly, several subgroup analyses (i.e., based on age, status of gout treatment, study design, the methodological quality score, and disease definitions) were performed to more thoroughly understand any possible association. However, we acknowledge that the present study also has several limitations, which must be considered when interpreting our findings. Firstly, we could not conduct a subgroup analysis according to the type of urate lowering drug, due to a lack of data. Nevertheless, a study by Cortese and colleagues [41] found that patients with gout who were treated with allopurinol had a lower risk of developing PD. Therefore, future research is needed to evaluate the association between gout and PD, in the light of urate lowering drugs that gout patients may be using. Secondly, publication bias was identified in our study, according to the funnel plots and Egger’s test. However, the validity of these methods in samples consisting of ten or fewer studies has been called into question [63]. Thirdly, a high degree of heterogeneity was detected in our analysis, which might affect confidence in our findings. Fourthly, while we retrieved the effect sizes that controlled for several confounders, observational studies are always prone to residual biases that might not have been considered in the adjustment process. Fifthly, the majority of the included studies had medical record-based designs, which implies the possibility of data misclassification and limited the ability to evaluate the associations according to more disease characteristics and demographic factors. Sixthly, although comprehensive database searches were undertaken to identify all eligible publications, we cannot dismiss the probability of missing unpublished data. Seventhly, although some studies used the International Classification of Disease criteria for gout, there is currently no universally accepted diagnostic criteria for gout. Therefore, the studies included in our meta-analysis were mainly comprised of patients with gout that was defined by clinical manifestations or using anti gout medications, which should be considered when interpreting the findings. Similarly, while some studies used the International Classification of Disease criteria for defining PD, several studies based their definition on the use of relevant drugs or the opinions of expert neurologists. Therefore, subgroup analyses based on gout and PD ascertainment method were undertaken.

## Conclusions

In contrast to several previous studies, which have found hyperuricemia to have a protective effect on the subsequent development of PD, our meta-analysis provides high-quality evidence that there is no overall association between gout and the risk of developing PD. In contrast, we found that females suffering from gout were more vulnerable to the subsequent onset of PD. Further observational investigations and meta-analyses, as well as more subgroup analyses of the demographic and behavioral risk factors (e.g. age, sex and smoking), are recommended in order to more clearly understand the association between gout and PD. In addition, the mechanism by which the sex differences occur, regarding the antioxidative and pro-oxidative features of uric acid, needs to be further investigated.  

## Supplementary Information

**Additional file 1:** **Supplementary Table 1.** Search strategies for PubMed, Scopus, Web of Science, and Google scholar. **Supplementary Table 2.** Comorbid conditions of individuals who participated in the studies included in the meta-analysis. **Supplementary Table 3. **Risk of bias assessment for the included cohort studies. **Supplementary Table4.** Risk of bias assessments for the included case-control studies.

## Data Availability

The data that support the findings of this study are available from the corresponding author upon reasonable request.
