# Supplementary material for: Association between gout and the development of Parkinson’s disease: a systematic review and meta-analysis
Source: BMC Neurol. 2022 Oct 11;22:383. doi: 10.1186/s12883-022-02874-0 (PMC9552480; doi:10.1186/s12883-022-02874-0)
Supplement: Supplementary file 1 — Additional file 1: Supplementary Table 1. Search strategies for PubMed, Scopus, Web of Science, and Google scholar. Supplementary Table 2. Comorbid conditions of individuals who participated in the studies included in the meta-analysis. Supplementary Table 3. Risk of bias assessment for the included cohort studies. Supplementary Table4. Risk of bias assessments for the included case-control studies. [file 12883_2022_2874_MOESM1_ESM.docx]

**Supplementary** **Table 1.** Search strategies for PubMed, Scopus, Web of Science, and Google scholar.

| Step | Search strategy | Results (search date: **16 March 2022**) |
| --- | --- | --- |
| PubMed | | |
| 1 | (((((((("Parkinson Disease"[Mesh]) OR "Parkinsonian Disorders"[Mesh]) OR (("Parkinson disease"[Title/Abstract]) AND ("Lewy body"[Title/Abstract]))) OR (("Parkinson disease"[Title/Abstract]) AND ("Lewy body"[Title/Abstract]))) OR ((((((((((((Parkinson Disease[Title/Abstract]) OR (Parkinsonian Disorders[Title/Abstract])) OR ("Parkinson’s disease"[Title/Abstract])) OR (Parkinsonism[Title/Abstract])) OR ("Idiopathic Parkinson Disease"[Title/Abstract])) OR ("Lewy Body Parkinson Disease"[Title/Abstract])) OR (Idiopathic[Title/Abstract])) OR (Lewy Body[Title/Abstract])) ) OR ("Primary Parkinsonism"[Title/Abstract])) OR ("Paralysis Agitans"[Title/Abstract])) OR (Parkinson[Title/Abstract]))) OR (("Parkinson disease"[Title/Abstract]) AND ("Lewy body"[Title/Abstract]))) OR (("Parkinson disease"[Title/Abstract]) AND (Idiopathic[Title/Abstract]))) OR ((Parkinsonism[Title/Abstract]) AND (Primary[Title/Abstract]))) OR ((Parkinson[Title/Abstract]) AND (disease[Title/Abstract])) | 269,910 |
| 2 | ((("Gout"[Mesh]) OR "Hyperuricemia"[Mesh]) OR "Uric Acid"[Mesh]) OR (((((((gout*[Title/Abstract]) OR (hyperuricemia*[Title/Abstract])) OR (tophus*[Title/Abstract])) OR (tophi*[Title/Abstract])) OR (tophaceous*[Title/Abstract])) OR (hyperuricaemia*[Title/Abstract])) OR ("uric acid"[Title/Abstract])) | 57,795 |
| 3 | #1 AND #2 | 899 |
|  | Scopus |  |
| 1 | TITLE-ABS-KEY ("Parkinson Disease") OR TITLE-ABS-KEY ("Parkinsonian Disorders") OR TITLE-ABS-KEY ("Parkinson's disease") OR TITLE-ABS-KEY (parkinsonism) OR TITLE-ABS-KEY ("Idiopathic Parkinson Disease") OR TITLE-ABS-KEY ("Lewy Body Parkinson Disease") OR TITLE-ABS-KEY ("Primary Parkinsonism") OR TITLE-ABS-KEY ("Paralysis Agitans") OR TITLE-ABS-KEY (Parkinson) | 199,140 |
| 2 | TITLE-ABS-KEY ( gout* ) OR TITLE-ABS-KEY ( hyperuricemia* ) OR TITLE-ABS-KEY ( hyperuricaemia* ) OR TITLE-ABS-KEY ( tophus* ) OR TITLE-ABS-KEY ( tophi* ) OR TITLE-ABS-KEY ( tophaceous* ) OR TITLE-ABS-KEY ( "uric acid" ) | 96,351 |
| 3 | #1 AND #2 | 730 |
|  | Web of Science |  |
| 1 | ((((((((TS=("Parkinson's disease")) OR TS=(Parkinsonism)) OR TS=("Parkinsonian disorders")) OR TS=("idiopathic Parkinson disease")) OR TS=("Lewy body Parkinson disease")) OR TS=("Parkinson disease")) OR TS=("Primary Parkinsonism")) OR TS=("Paralysis Agitans")) OR TS=(Parkinson) | 161,261 |
| 2 | ((((((TS=(gout*)) OR TS=(hyperuricemia*)) OR TS=(hyperuricaemia)) OR TS=(tophus*)) OR TS=(tophi*)) OR TS=(tophaceous*)) OR TS=("uric acid") | 55,956 |
| 3 | #1 AND #2 | 503 |
|  | Google Scholar | Search data: 3 June 2022 |
| 1 | ("Parkinson disease" OR Parkinsonism OR "Parkinsonian disorders" OR "idiopathic Parkinson disease" OR "Lewy body Parkinson disease" OR "Parkinson disease" OR "Primary Parkinsonism" OR "Paralysis Agitans" OR Parkinson) AND (gout* OR hyperuricemia* OR hyperuricaemia* OR tophi* OR tophus* OR tophaceous* OR “uric acid”) | 27,200 |

**Supplementary Table 2.** Comorbid conditions of individuals who participated in the studies included in the meta-analysis

| **Study ID** | Alonso et al. 2007 (1) | Cortese et al. 2018 (2) | De Vera et al. 2008 (3) | Hu et al. 2020 (4) | Kim et al. 2021 (5) | Lai et al. 2014 (6) | Pakpoor et al. 2015 (7) | Schernhammer et al. 2013 (8) | Singh et al. 2019 (9) | Pou et al. 2022 (10) |
| --- | --- | --- | --- | --- | --- | --- | --- | --- | --- | --- |
| **Smoking** | Case: 25.6%  Control: 33.7% | N/A | N/A | N/A | N/A | Case: 14 (0.36%)  Control: 48 (0.31%) | N/A | N/A | N/A | Case: 1,516 (8.6%)  Control: 7,392 (10.5%) |
| **Alcoholism** | N/A | N/A | N/A | N/A | N/A | Case: 12  Control: 22 | N/A | N/A | N/A | N/A |
| **Hypertension** | N/A | N/A | Case: 4,711 (41.9%)  Control: 15,879 (28.3%) | Case: 3,240 (24.3%)  Control: 1,887 (9.8%) | Case: 211,097 (64.52%)  Control: 140,890 (43.06%) | Case: 3,145  Control: 10,143 | N/A | N/A | Total: 833,128 (48.3%) | Total: 51,401 (58.3%)  Case: 10,572 (60.0%)  Control: 40,829 (57.9%) |
| **Diabetes** | N/A | N/A | Case: 1,173 (10.4%)  Control: 4,413 (7.9%) | Case: 1,664 (9.6%)  Control: 950 (4.5%) | Case: 186,198 (56.91%)  Control: 130,351  (39.84%) | Case: 1,446  Control: 4,186 | N/A | N/A | Total: 319,089 (18.5%) | Total: 18692 (21.2%)  Case: 4,208 (23.9%)  Control: 14484 (20.5%) |
| **Hyperlipidemia** | N/A | N/A | Case: 1,143 (10.2%)  Control: 4,345 (7.7%) | N/A | N/A | Case: 1,175  Control: 3,920 | N/A | N/A | Total: 601,188 (34.8%) | N/A |
| **Peripheral Vascular Disease** | N/A | N/A | N/A | N/A | N/A | N/A | N/A | N/A | Total: 167,945 (9.7%) | N/A |
| **Cerebrovascular disease** | N/A | N/A | N/A | Case: 1,147 (6.9%)  Control: 829 (3.7%) | N/A | Case: 2,237  Control: 3,760 | N/A | N/A | Total: 167,247 (9.7%) | N/A |
| **Autoimmune disease** | N/A | N/A | N/A | Case: 316 (2.2%)  Control: 163 (1.2%) | N/A | N/A | N/A | N/A | N/A | N/A |
| **AIDS** | N/A | N/A | N/A | N/A | N/A | N/A | N/A | N/A | Total: 547 (0.03%) | N/A |
| **Dyslipidemia** | N/A | N/A | N/A | Case: 2,230 (14.0%)  Control: 1,000 (4.8%) | Case: 284,794 (87.05%)  Control: 203,823 (62.3%) | N/A | N/A | N/A | N/A | Total: 36,982 (42.0%)  Case: 7,322 (41.5%)  Control: 29,660 (42.1%) |
| **Nephropathy** | N/A | N/A | N/A | Case: 1,219 (5.9%)  Control: 661 (2.8%) | N/A | N/A | N/A | N/A | Total: 59,340 (3.4%) | N/A |
| **COPD** | N/A | N/A | Case: 2,584 (23.0%)  Control: 10,295 (18.3%) | N/A | N/A | N/A | N/A | N/A | N/A | N/A |
| **Connective Tissue Disease** | N/A | N/A | N/A | N/A | N/A | N/A | N/A | N/A | Total: 47,999 (2.8%) | N/A |
| **Stroke** | N/A | N/A | N/A | N/A | Case: 34,920 (10.67%)  Control: 27,777 (8.49%) | N/A | N/A | N/A | N/A | N/A |
| **Dementia** | N/A | N/A | N/A | N/A | N/A | Case: 559  Control: 343 | N/A | N/A | Total: 75,936 (4.4%) | N/A |
| **Chronic liver disease** | N/A | N/A | N/A | Case: 2,713 (19.4%)  Control: 1,638 (8.7%) | N/A | N/A | N/A | N/A | N/A | N/A |
| **Chronic lung disease** | N/A | N/A | N/A | Case: 989 (4.9%)  Control: 684 (1.9%) | N/A | N/A | N/A | N/A | Total: 269,751 (15.6%) | N/A |
| **Ischemic heart disease** | N/A | N/A | N/A | N/A | Case: 102,591  (31.36%)  Control: 73,162 (22.36%) | N/A | N/A | N/A | N/A | N/A |
| **Coronary Artery Disease** | N/A | N/A | N/A | N/A | N/A | N/A | N/A | N/A | Total: 302,982 (17.6%) | N/A |
| **Obesity** | N/A | N/A | N/A | N/A | N/A | Case: 13  Control: 42 | N/A | N/A | N/A | N/A |
| **Chronic Kidney Disease** | N/A | N/A | N/A | N/A | N/A | Case: 297  Control: 722 | N/A | N/A | N/A | N/A |
| **Major Depressive Disorder** | N/A | N/A | N/A | N/A | N/A | case: 571  Control: 787 | N/A | N/A | N/A | N/A |
| **Hemiplegia** | N/A | N/A | N/A | N/A | N/A | N/A | N/A | N/A | Total: 14,118 (0.82%) | N/A |
| **Head Injury** | N/A | N/A | N/A | N/A | N/A | Case: 248  Control: 524 | N/A | N/A | N/A | N/A |
| **Myocardial infarction** | N/A | N/A | N/A | N/A | N/A | N/A | N/A | N/A | Total: 68,537 (4.0%) | N/A |
| **Heart Failure** | N/A | N/A | N/A | N/A | N/A | N/A | N/A | N/A | Total: 202,196 (11.7%) | N/A |
| **Peptic Ulcer Disease** | N/A | N/A | N/A | N/A | N/A | N/A | N/A | N/A | Total: 32,624 (1.9%) | N/A |
| **Polypharmacy** | N/A | N/A | N/A | N/A | N/A | Case: 1,589  Control: 3,313 | N/A | N/A | N/A | N/A |

**Supplementary Table 3.** Risk of bias assessment for the included cohort studies.

|  | **DOP** | **1.Representativeness of the exposed cohort:** | **2. Selection of the non-exposed cohort:** | **3.Ascertainment of exposure:** | **4.Demonstration that outcome of interest was not present at start of study:** | **5.Comparability of cohorts on the basis of the design or analysis:** | **6.Assessment of outcome:** | **7. Was follow-up long enough for outcomes to occur:** | **8. Adequacy of follow up of cohorts** | **Overall score** |
| --- | --- | --- | --- | --- | --- | --- | --- | --- | --- | --- |
| Cortese et al. (2) | 2018 | A | A | A | A | A | B | A | D | 7 |
| De Vera et al. (3) | 2008 | A | A | A | A | A | B | A | D | 7 |
| Hu et al. (4) | 2020 | A | A | A | A | A | B | A | D | 7 |
| Kim et al. (5) | 2021 | A | A | A | B | A | B | A | D | 6 |
| Pakpoor et al. (7) | 2015 | A | A | A | B | A | B | A | D | 6 |
| Singh et al. (9) | 2019 | B | A | A | B | A | B | A | D | 6 |

Abbreviations: DOP: date of publication

Note: A study can be awarded a maximum of one star for each numbered item within the Selection and Outcome categories. A maximum of two stars can be given for Comparability

**Selection**

1) Representativeness of the exposed cohort

a) truly representative of the average _______________ (describe) in the community *

b) somewhat representative of the average ______________ in the community *

c) selected group of users e.g. nurses, volunteers

d) no description of the derivation of the cohort

2) Selection of the non-exposed cohort

a) drawn from the same community as the exposed cohort *

b) drawn from a different source

c) no description of the derivation of the non-exposed cohort

3) Ascertainment of exposure

a) secure record (e.g. surgical records) *

b) structured interview *

c) written self-report

d) no description

4) Demonstration that outcome of interest was not present at start of study

a) yes *

b) no

**Comparability**

1) Comparability of cohorts on the basis of the design or analysis

a) study controls for _____________ (select the most important factor) *

b) study controls for any additional factor * (This criteria could be modified to indicate specific control for a second important factor.)

**Outcome**

1) Assessment of outcome

a) independent blind assessment *

b) record linkage *

c) self-report

d) no description

2) Was follow-up long enough for outcomes to occur

a) yes (select an adequate follow up period for outcome of interest) *

b) no

3) Adequacy of follow up of cohorts

a) complete follow up - all subjects accounted for *

b) subjects lost to follow up unlikely to introduce bias - small number lost - > ____ % (select an adequate %) follow up, or description provided of those lost) *

c) follow up rate < ____% (select an adequate %) and no description of those lost

d) no statement

**Supplementary Table 4.** Risk of bias assessments for the included case-control studies.

|  | **DOP** | **1. Is the case definition adequate?** | **2.Representativeness of the cases** | **3. Selection of Controls** | **4. Definition of Controls** | **5. Comparability of cases and controls on the basis of the design or analysis** | **6. Ascertainment of exposure** | **7. Same method of ascertainment for cases and controls** | **8. Non-Response rate** | **Overall score** |
| --- | --- | --- | --- | --- | --- | --- | --- | --- | --- | --- |
| Alonso et al. (1) | 2007 | A | A | A | B | A | A | A | C | 6 |
| Lai et al. (6) | 2014 | A | B | A | A | A | A | A | C | 6 |
| Schernhammer et al. (8) | 2013 | A | B | A | A | A | A | A | C | 6 |
| Pou et al. (10) | 2022 | A | B | A | A | A | A | A | C | 6 |

Abbreviations: DOP: date of publication.

Note: A study can be awarded a maximum of one star for each numbered item within the Selection and

Exposure categories. A maximum of two stars can be given for Comparability.

**Selection**

1) Is the case definition adequate?

a) yes, with independent validation *

b) yes, e.g. record linkage or based on self-reports

c) no description

2) Representativeness of the cases

a) consecutive or obviously representative series of cases *

b) potential for selection biases or not stated

3) Selection of Controls

a) community controls *

b) hospital controls

c) no description

4) Definition of Controls

a) no history of disease (endpoint) *

b) no description of source

**Comparability**

1) Comparability of cases and controls on the basis of the design or analysis

a) study controls for _______________ (Select the most important factor.) *

b) study controls for any additional factor * (This criteria could be modified to indicate specific control for a second important factor.)

**Exposure**

1) Ascertainment of exposure

a) secure record (e.g. surgical records) *

b) structured interview where blind to case/control status *

c) interview not blinded to case/control status

d) written self-report or medical record only

e) no description

2) Same method of ascertainment for cases and controls

a) yes *

b) no

3) Non-Response rate

a) same rate for both groups *

b) non respondents described

c) rate different and no designation

**References**

1. Alonso A, Garcia Rodriguez LA, Logroscino G, Hernan MA. Gout and risk of Parkinson disease - A prospective study. Neurology. 2007;69(17):1696-700.

2. Cortese M, Riise T, Engeland A, Ascherio A, Bjørnevik K. Urate and the risk of Parkinson's disease in men and women. Parkinsonism Relat Disord. 2018;52:76-82.

3. De Vera M, Rahman MM, Rankin J, Kopec J, Gao X, Choi H. Gout and the risk of Parkinson's disease: a cohort study. Arthritis Rheum. 2008;59(11):1549-54.

4. Hu LY, Yang AC, Lee SC, You ZH, Tsai SJ, Hu CK, et al. Risk of Parkinson's disease following gout: a population-based retrospective cohort study in Taiwan. BMC Neurol. 2020;20(1):338.

5. Kim JH, Choi IA, Kim A, Kang G. Clinical Association between Gout and Parkinson's Disease: A Nationwide Population-Based Cohort Study in Korea. Medicina (Kaunas). 2021;57(12).

6. Lai SW, Lin CH, Lin CL, Liao KF. Gout and Parkinson's Disease in Older People: An Observation in Taiwan. International Journal of Gerontology. 2014;8(3):166-7.

7. Pakpoor J, Seminog OO, Ramagopalan SV, Goldacre MJ. Clinical associations between gout and multiple sclerosis, Parkinson's disease and motor neuron disease: record-linkage studies. BMC Neurol. 2015;15:16.

8. Schernhammer E, Qiu JH, Wermuth L, Lassen CF, Friis S, Ritz B. Gout and the risk of Parkinson's disease in Denmark. European Journal of Epidemiology. 2013;28(4):359-60.

9. Singh JA, Cleveland JD. Gout and the risk of Parkinson's disease in older adults: a study of U.S. Medicare data. BMC Neurol. 2019;19(1):4.

10. Pou MA, Orfila F, Pagonabarraga J, Ferrer-Moret S, Corominas H, Diaz-Torne C. Risk of Parkinson's disease in a gout Mediterranean population: A case-control study. Joint Bone Spine. 2022:105402.
